# Risk factors for intraocular pressure elevation in a six-month period after ab interno trabeculotomy using a Kahook Dual Blade

**DOI:** 10.1186/s12886-022-02545-1

**Published:** 2022-07-30

**Authors:** Setsu Murakami-Kojima, Eri Takahashi, Momoka Eguchi-Matsumoto, Junji Saruwatari, Kei-ichi Nakashima, Toshihiro Inoue

**Affiliations:** 1 Department of Ophthalmology, Faculty of Life Sciences, Kumamoto University, Kumamoto, Japan; 2 Department of Pharmacology and Therapeutics, Graduate School of Pharmaceutical Sciences, Kumamoto University, Kumamoto, Japan

**Keywords:** Trabeculotomy ab interno, Kahook dual blade, Primary open angle glaucoma, Exfoliation glaucoma, Intraocular pressure

## Abstract

**Background:**

To examine the risk factors for an early postoperative intraocular pressure (IOP) increase after ab interno trabeculotomy using a Kahook Dual Blade (KDB trabeculotomy).

**Methods:**

A retrospective study was performed in 76 exfoliation glaucoma (EXG) eyes and 56 primary open angle glaucoma (POAG) eyes that underwent KDB trabeculotomy, with or without cataract surgery at Kumamoto University Hospital. Postoperative high IOP was classified as IOP≥20 mmHg (within three months after surgery, whether persistent or temporary), transient IOP≥20 mmHg (IOP≥20 mmHg after surgery, then dropped below 20 mmHg), and the presence of IOP spikes (≥ 10 mmHg from baseline). Risk factors were examined using logistic regression analysis.

**Results:**

The preoperative mean IOP (SD) was 24.98 (7.23) mmHg in patients with EXG and 21.28 (6.58) mmHg in patients with POAG. IOP was reduced by 32.1% in patients with EXG and by 17.7% in patients with POAG at 6 months after surgery. Postoperative IOP≥20 mmHg was observed in 56.6% of EXG patients and in 51.8% of POAG patients. IOP spikes occurred in 15.8% of EXG patients and in 14.3% of POAG patients. Logistic regression analysis showed that factors with significant odds ratios (ORs) were age (OR = 0.866, 95% CI = 0.793–0.945), preoperative medication use (OR = 2.02, 95% CI = 1.17–3.49), trabeculotomy in combination with cataract surgery (OR = 0.0674, 95% CI = 0.015–0.303), and IOP at day 1 (OR = 1.41, 95% CI = 1.18–1.68) for postoperative IOP≥20 mmHg, the IOP at day 1 (OR = 1.1, 95% CI = 1.03–1.17) for transient IOP≥20 mmHg, and age (OR = 0.948, 95% CI = 0.901–0.997) and preoperative IOP (OR = 0.83, 95% CI = 0.736–0.936) for IOP spikes.

**Conclusion:**

Although KDB trabeculotomy is an effective treatment for patients with EXG and POAG, patients who take multiple preoperative medications and have a high IOP on day 1 require careful follow-up to prevent postoperative IOP elevation.

**Supplementary Information:**

The online version contains supplementary material available at 10.1186/s12886-022-02545-1.

## Key messages

Trabeculotomy ab interno using a Kahook dual blade (KDB trabeculotomy) is an effective treatment for patients with primary open angle glaucoma and exfoliation glaucoma.

The number of preoperative glaucoma medications and IOP on postoperative day 1 were associated with an increase in postoperative IOP≥20 mmHg.

The risk of an increase in postoperative IOP≥20 mmHg was lower in elderly patients and in patients who underwent combined cataract surgery.

Our results may be helpful in the postoperative management of KDB trabeculotomy.

## Background

Glaucoma is a disease that progressively impairs the patient’s visual field, causes blindness and requires treatment to lower intraocular pressure (IOP) [1]. The aim of glaucoma surgery is to lower IOP and avoid long-term medication use. Minimally invasive glaucoma surgery (MIGS) is a new surgical technique, while ab interno trabeculotomy is a less invasive method that has a shorter operation time in comparison to conventional methods of creating scleral flaps, such as trabeculectomy and trabeculotomy ab externo [2]. Although some methods for ab interno trabeculotomy have been developed, these procedures excise or incise the diseased trabecular meshwork (TM) under gonioscopy in patients whose angle is open [3–5]; the Kahook Dual Blade (KDB) resects the TM from the anterior chamber. Seibold et al. preclinically evaluated three MIGS devices, the microvitreoretinal blade, the Trabectome, and KDB. KDB stripped 157.5 ± 26.3 degree-wide TM tissues and achieved IOP reduction from 18.3 ± 3.0 to 11.2 ± 2.2 mmHg as well as others in a human eye perfusion model [4].

An increasing number of reports associated with MIGS have identified the risk factors and prognosis; however, there are still problems to be solved. A vexing complication in procedures targeting the TM tissue is IOP spikes, and the incidence of IOP spikes in ab interno trabeculotomy using a KDB (KDB trabeculotomy) ranges from 7.7% to 18.2% [6–9]. Uncontrolled high IOP levels may worsen visual field defects; one report showed that 14.3% (6/42) of eyes required additional procedures for IOP reduction within three months after KDB trabeculotomy [10]. In this study, we present the 6-month IOP transition after KDB trabeculotomy in EXG and POAG patients and assess the risk factors for high IOP after surgery.

## Methods

### Patients

The study was performed in accordance with the Ethical Guidelines for Medical and Health Research Involving Human Subjects in Japan as well as the Declaration of Helsinki. Adult patients with EXG or POAG who were followed up for more than 3 months after KDB trabeculotomy were included in this study. EXG was diagnosed based on findings of exfoliation materials at the edge of the pupil, on the lens surface or in the angle as Sampaolesi’s line. POAG was diagnosed based on findings of a normal angle appearance without other underlying causes of IOP elevation, the appearance of characteristic optic discs, and a correlation between thinning of the retinal nerve fibre layer and visual field defects. The surgical indication was determined by two or more glaucoma surgeons, and KDB trabeculotomy was performed by three glaucoma surgeons at Kumamoto University Hospital between April 2018 and November 2019. According to the guidelines of Glaucoma Surgery Trials from the World Glaucoma Association, IOP was measured by Goldmann applanation tonometry, and the baseline IOP was determined as the mean of three IOP levels measured before KDB trabeculotomy. IOP spike was defined as an IOP increase≥10 mmHg from baseline.

### Surgical techniques and postoperative management

The glaucoma surgeons followed the standard techniques for KDB trabeculotomy [6]. First, temporal corneal incisions were made using a 20-gauge ophthalmic knife, and the anterior chamber was filled with viscoelastic sodium hyaluronate. The patient’s head was tilted away, and the microscope was tilted towards the surgeon. Then, a gonioprism was set on the cornea to view the nasal TM. The KDB was inserted through a temporal corneal incision, and approximately 120 degrees of nasal TM was stripped and was removed by intraocular forceps. After removal of the sodium hyaluronate, the wounds were hydrated to close the corneal incision. During combined KDB trabeculotomy with cataract surgery, a TM excision procedure was made after continuous curvilinear capsulorhexis (CCC). Cataract surgery was performed based on the following procedure. In brief, first, one 2.4-mm and two 1.3-mm temporal corneal incisions were made, and CCC and hydrodissection were performed. The nucleus and cortex of the lens were removed by phacoemulsification (phaco) and aspiration. After a foldable intraocular lens was implanted into a capsular bag filled with viscoelastic sodium hyaluronate, viscoelastic sodium hyaluronate was completely aspirated, and the wounds were hydrated to close the incisions. At the end of surgery, 0.1% betamethasone was subconjunctivally injected. Postoperatively, antibiotics and 0.1% betamethasone sodium phosphate eye drops were started and applied four times daily. Nonsteroidal anti-inflammatory drops were used twice daily in patients undergoing KDB trabeculotomy combined with phaco. These treatments were tapered based on inflammation levels. All hypotension medications were stopped on the day of the operation and restarted based on IOP levels and the extent of visual field defects.

### Statistical analysis

The data are expressed as the mean (standard deviation) or proportion for categorical variables. Continuous and categorical variables were compared between groups by Student’s *t test*, paired *t test* or one-way analysis of variance and Fisher’s exact test. The odds ratio (OR) and 95% confidence interval (CI) for the risk of IOP elevation after surgery were calculated using a multivariable logistic regression model incorporating age, sex, diagnosis (EXG or POAG), preoperative IOP, number of medications used before the operation, trabeculotomy combined with phaco, IOP level on the day after surgery, and hyphema as covariates. A *p* value < 0.05 was considered statistically significant. Multiple comparisons were corrected using Bonferroni’s method, and values of *P* < 0.05/n were considered statistically significant after correction for the number of comparisons made. The statistical analyses were performed using R software, version 3.5.0 (R Foundation for Statistical Computing, Vienna, Austria).

## Results

Table 1 shows the characteristics and baseline glaucoma status. Data were collected from 132 eyes of 120 patients, all of whom were Japanese ethnicity. A total of 57.6% of eyes had EXG, and 42.4% had POAG. Table 2 shows the characteristics and baseline glaucoma data of the EXG and POAG groups. There were significant differences in age (*p* = 0.003) and the following ocular parameters between the EXG and POAG groups. The mean IOP at baseline in patients with EXG was higher than that in patients with POAG (*p* = 0.003), and 31 eyes with EXG previously underwent phaco (*p* < 0.001); therefore, there were more EXG patients with pseudophakic eyes than those in the POAG group among patients who underwent KDB trabeculotomy alone.

**Table 1:** Patient demographics and baseline ocular parameters

	Total (132 eyes of 120 patients)
Sex [n (%)]	
Female	57 (43.2)
Male	75 (56.8)
Age (y)	
Mean ± SD	76.7 ± 10.4
Range	21–94
Glaucoma diagnosis [n (%)]	
EXG	76 (57.6)
POAG	56 (42.4)
Previous intraocular surgery	
Cataract surgery [n (%)]	33 (25.0)
Filtration surgery [n (%)]	7 (5.3)
Trabeculotomy ab externo [n (%)]	4 (3.0)
Preoperative status	
IOP at baseline (mmHg)	
Mean ± SD	23.4 ± 7.2
Range	12–47
No. glaucoma medicatons at baseline	
Mean ± SD	4.0 ± 2.8
Combined phaco + KDB [n (%)]	68 (51.5)
Standalone KDB [n (%)]	64 (48.5)

*POAG* primary open-angle glaucoma, *EXG* exfoliation glaucoma, *IOP* intraocular pressure, *KDB* Kahook Dual Blade, *phaco* phacoemulsification

**Table 2:** Demographics of patients with EXG and POAG

	EXG (*n* = 76)	POAG (*n* = 56)	*P* value
Sex			
Female	34	23	0.724*
Age (y)			
Mean ± SD	78.9 ± 8.31	73.6 ± 12.1	0.003
Range	50–94	21–91	
Previous intraocular surgery			
Cataract surgery	31	2	< 0.001*
Filtration surgery	4	3	1*
Trabeculotomy ab externo	4	0	0.137*
Preoperative status			
IOP at baseline (mmHg)			
Mean ± SD	25.0 ± 7.2	21.3 ± 6.6	0.003
Range	12–46	12–47	
No. glaucoma medications			
Mean ± SD	4.3 ± 3.5	3.7 ± 1.1	0.226
Combined phaco + KDB	32	36	
Standalone KDB			
Pseudophakia	31	2	< 0.001*
Phakia	13	18	

Data are the mean (standard deviation) or proportion for categorical variables

*POAG* primary open angle glaucoma, *EXG* exfoliation glaucoma, *IOP* intraocular pressure, *phaco* phacoemulsification, *KDB* Kahook Dual Blade

^*^Analysed by Fisher’s exact test, otherwise Student’s t test

### IOP Reduction

The mean postoperative IOP and the mean change from baseline in the EXG and POAG groups are shown in Table 3. The mean postoperative IOP in the EXG group was significantly reduced at all visits, while that in the POAG group was significantly reduced except at week 1 and month 1 after surgery.

**Table 3:** Reduction of mean intraocular pressure at each time point after surgery

	EXG		
	IOP (mmHg)	Mean change from baseline ± SD	*P* value*
	Mean ± SD	(Ratio of mean change)	
Baseline	24.98 ± 7.23	Reference	
Day 1	17.26 ± 7.46	7.71 ± 10.46 (-30.9)	< 0.001
Week 1	21.04 ± 8.71	3.94 ± 11.51 (-15.8)	0.004
Month 1	19.43 ± 7.14	5.54 ± 8.64 (-22.2)	< 0.001
Month 3	16.64 ± 4.90	8.33 ± 8.72 (-33.4)	< 0.001
Month 6	17.03 ± 6.98	8.01 ± 9.12 (-32.1)	< 0.001
	POAG		
	IOP (mmHg)	Mean change from baseline ± SD	*P* value*
	Mean ± SD	(Ratio of mean change)	
Baseline	21.28 ± 6.58	Reference	
Day 1	16.71 ± 6.61	4.56 ± 8.24 (-21.4)	< 0.001
Week 1	19.07 ± 6.71	2.21 ± 7.44 (-10.4)	0.031
Month 1	20.30 ± 7.99	0.97 ± 7.83 (-4.57)	0.356
Month 3	17.30 ± 3.41	3.97 ± 6.32 (-18.7)	< 0.001
Month 6	17.41 ± 3.67	3.77 ± 7.15 (-17.7)	< 0.001

*POAG* primary open angle glaucoma, *EXG* exfoliation glaucoma, *IOP* intraocular pressure

^*^P values were analysed by paired *t test*, and multiple comparisons were corrected using Bonferroni’s method, *i.e.*, values of *P* < 0.05/4 were considered to be statistically significant

**Fig. 1:**
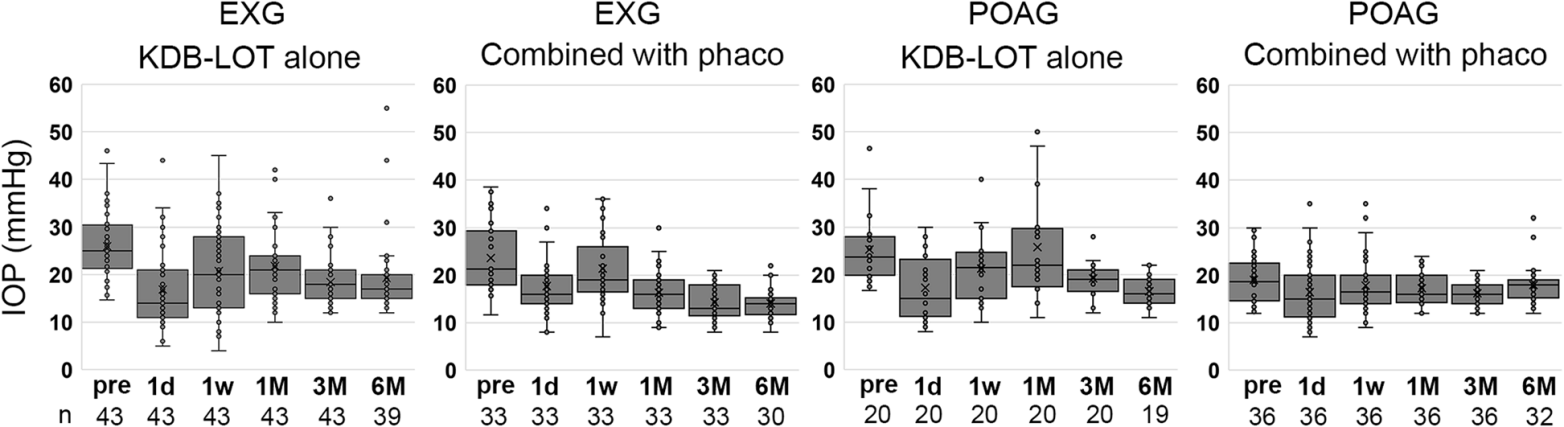
IOP distributions at different times after trabeculotomy ab interno using a Kahook dual blade. Trabeculotomy ab interno using a Kahook dual blade (KDB-LOT) alone or combined with phacoemulsification (phaco) was performed in patients with exfoliation glaucoma (EXG) or primary open angle glaucoma (POAG). The boxes represent the 25th, 50th, and 75th percentiles of IOP and cross marks show the average

**Fig. 2:**
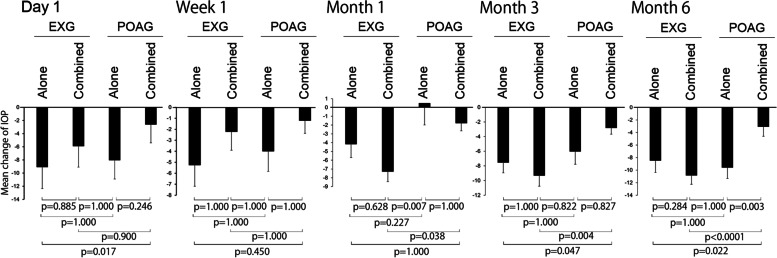
Changes in IOP after trabeculotomy ab interno using a Kahook dual blade. Mean change in intraocular pressure (IOP) with standard error bars for patients with exfoliation glaucoma (EXG) or primary open-angle glaucoma (POAG) undergoing KDB trabeculotomy with (combined) or without (alone) phacoemulsification. Statistical analysis was performed using one-way analysis of variance with Bonferroni correction

The patients were further categorized by the presence or absence of phaco; EXG-alone, EXG-combined, POAG-alone, and POAG-combined. The variation in postoperative IOP was visually greater with KDB trabeculotomy alone than with the combined procedure (Fig. 1). The IOP of the EXG-combined group at month 6 was the lowest among the four groups, and the mean number of medications was also the lowest (Tables 4 and 5). Figure 2 shows the mean change in IOP among the four groups. The EXG-alone group tended to have the greatest rate of IOP reduction on day 1. At month 1, the IOP was lower in the EXG-combined group than in the POAG group (POAG-alone, *p* = 0.007 and POAG-combined, *p* = 0.038). At month 3, the IOP reduction rate was lower in the EXG (EXG-alone, *p* = 0.047 and EXG-combined, *p* = 0.004) than in the POAG-combined group. The rate of IOP reduction at month 6 was significantly lower in the POAG-combined group than in the other groups (Fig. 2).

**Table 4:** Reduction of mean intraocular pressure at each time point after surgery in EXG

	KDB alone			
	IOP (mmHg)	Number of med. ± SD	Mean change from baseline ± SD	*P* value*
	Mean ± SD		(Ratio of mean change)	
Baseline	26.00 ± 7.25	4.86 ± 4.45	Reference	
Day 1	16.91 ± 8.35	0	9.09 ± 10.67 (-35.0)	< 0.001
Week 1	20.74 ± 9.51	0.93 ± 1.28	5.26 ± 12.68 (-20.2)	0.01
Month 1	21.91 ± 7.89	1.98 ± 1.18	4.19 ± 9.86 (-16.1)	0.008
Month 3	18.44 ± 5.01	2.70 ± 1.15	7.56 ± 9.12 (-29.1)	< 0.001
Month 6	19.31 ± 8.09	2.74 ± 1.27	6.34 ± 9.82 (-24.4)	< 0.001
	KDB combined with phaco			
	IOP (mmHg)	Number of med. ± SD	Mean change from baseline ± SD	*P* value*
	Mean ± SD		(Ratio of mean change)	
Baseline	23.64 ± 7.08	3.55 ± 1.48	Reference	
Day 1	17.73 ± 6.21	0	5.91 ± 10.05 (-25.0)	0.002
Week 1	21.42 ± 7.65	1.22 ± 1.18	2.22 ± 9.70 (-9.39)	0.198
Month 1	16.33 ± 4.48	1.78 ± 1.21	7.31 ± 6.45 (-30.9)	< 0.001
Month 3	14.30 ± 3.64	1.78 ± 1.31	9.34 ± 8.19 (-39.5)	< 0.001
Month 6	14.07 ± 3.54	1.50 ± 1.33	10.17 ± 7.74 (-43.0)	< 0.001

*EXG* exfoliation glaucoma, *IOP* intraocular pressure, *KDB* Kahook Dual Blade, *med.* medications

^*^P values were analysed by paired *t test*, and multiple comparisons were corrected using Bonferroni’s method, *i.e.*, values of *P* < 0.05/4 were considered to be statistically significant

**Table 5:** Reduction of mean intraocular pressure at each time point after surgery in POAG

	KDB alone			
	IOP (mmHg)	Number of med. ± SD	Mean change from baseline ± SD	*P* value*
	Mean ± SD		(Ratio of mean change)	
Baseline	25.30 ± 7.31	3.75 ± 1.02	Reference	
Day 1	17.25 ± 7.03	0	8.05 ± 8.13 (-31.8)	< 0.001
Week 1	21.30 ± 7.48	1.25 ± 1.48	4.00 ± 8.19 (-15.8)	0.042
Month 1	25.80 ± 10.69	2.15 ± 2.03	0.50 ± 11.12 (1.98)	0.084
Month 3	19.25 ± 4.05	2.65 ± 1.69	6.05 ± 7.74 (-23.9)	0.002
Month 6	16.53 ± 3.12	3.16 ± 1.42	8.84 ± 6.90 (-34.9)	< 0.001
	KDB combined with phaco			
	IOP (mmHg)	Number of med. ± SD	Mean change from baseline ± SD	*P* value*
	Mean ± SD		(Ratio of mean change)	
Baseline	19.04 ± 4.97	3.67 ± 1.12	Reference	
Day 1	16.42 ± 6.46	0	2.63 ± 7.75 (-13.8)	0.05
Week 1	17.93 ± 5.99	0.56 ± 1.05	1.21 ± 6.92 (-6.36)	0.302
Month 1	17.25 ± 3.38	1.11 ± 1.53	1.79 ± 5.22 (-9.40)	0.047
Month 3	16.22 ± 2.44	1.32 ± 1.45	2.82 ± 5.14 (-14.8)	0.002
Month 6	17.94 ± 3.92	1.63 ± 1.56	0.76 ± 5.44 (-23.9)	0.432

*POAG* primary open angle glaucoma, *IOP* intraocular pressure, *KDB* Kahook Dual Blade, *phaco* phacoemulsification, *med.* medications

^*^P values were analysed by paired *t test*, and multiple comparisons were corrected using Bonferroni’s method, *i.e.*, values of *P* < 0.05/4 were considered to be statistically significant

### IOP elevation and risk factors after KDB trabeculotomy

**Fig. 3:**
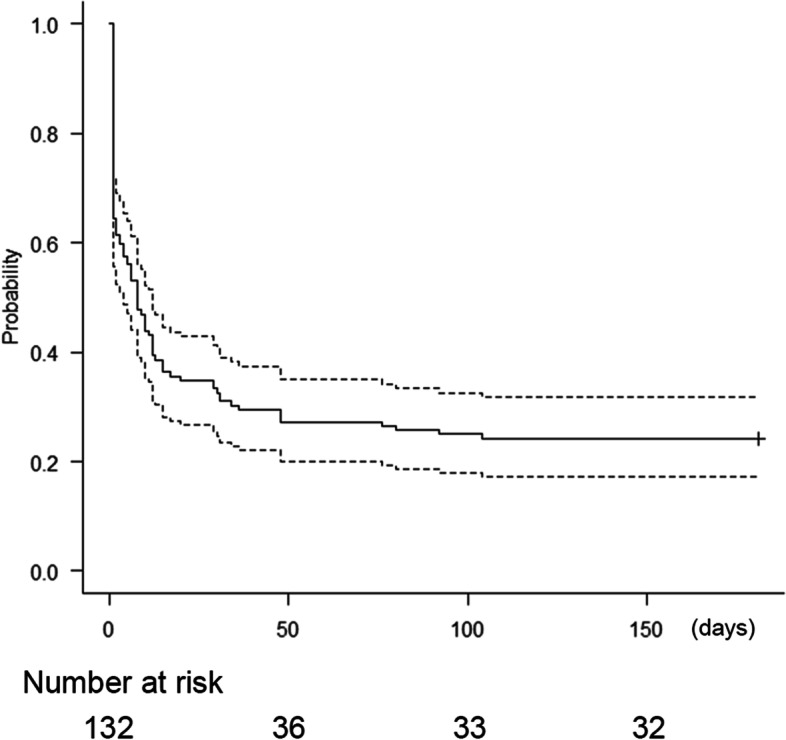
Kaplan–Meier curve for all eyes for the onset of IOP≥20 mmHg after surgery. Dotted lines indicate the 95% confidence intervals

The Kaplan–Meier curve in Fig. 3 shows the time points when the IOP≥20 mmHg postoperatively. The IOP levels of 17 eyes with EXG (22.4%) and 11 eyes with POAG (19.6%) were persistently≥20 mmHg at all visits. Transient IOP elevations of≥20 mmHg for 3 months were observed in 43 eyes with EXG (56.6%) and 29 eyes with POAG (51.8%). IOP spikes occurred in 12 eyes with EXG (15.8%) and in 8 eyes with POAG (14.3%). Hyphema occurred in 15 eyes, and two eyes were subjected to anterior chamber wash out due to massive postoperative hyphema. Two eyes underwent micropulse transscleral cyclophotocoagulation, and one eye underwent trabeculectomy within three months after surgery. Table 6 shows the results of the logistic regression analysis for prognostic factors of IOP elevation after surgery. Every 1-year increment in age (OR = 0.866, 95% CI = 0.793–0.945) and combined cataract surgery (OR = 0.0674, 95% CI = 0.015–0.303) significantly reduced the risk of IOP≥20 mmHg, while preoperative medication numbers (OR = 2.02, 95% CI = 1.17–3.49) and higher IOP at day 1 (OR = 1.41, 95% CI = 1.18–1.68) significantly increased this risk.

**Table 6:** Results of logistic regression model analysis for risk factors for intraocular pressure elevation

	≥ 20 mmHg			Transient ≥ 20 mmHg			IOP spike		
Factors	OR	95% CI	*P* value	OR	95% CI	*P* value	OR	95% CI	*P* value
Age (as 1-year increase)	0.866	0.793–0.945	0.001	0.975	0.937–1.01	0.206	0.95	0.901–0.997	0.004
Male sex (vs. female)	1.39	0.412–4.71	0.594	0.828	0.392–1.75	0.621	1.18	0.399–3.5	0.762
Diagnosis as POAG (vs. EXG)	0.864	0.251–2.98	0.817	0.766	0.345–1.7	0.521	0.57	0.18–1.77	0.328
Preoperative IOP	1.06	0.963–1.17	0.235	1.03	0.969–1.09	0.379	0.83	0.736–0.936	0.002
(as a 1 mmHg increase)									
Preoperative medications	2.02	1.17–3.49	0.012	0.955	0.839–1.09	0.487	1.02	0.851–1.22	0.823
(as a one-medication increase)									
KDB combined with phaco	0.0674	0.015–0.303	< 0.001	1.19	0.544–2.62	0.658	0.35	0.102–1.19	0.093
(vs. without phaco)									
IOP at day 1	1.41	1.18–1.68	< 0.001	1.1	1.03–1.17	0.003	1.08	0.998–1.17	0.056
(as a 1-mmHg increase)									
Hyphema (vs. absent)	0.569	0.0679–4.77	0.603	1.1	0.341–3.58	0.869	0.81	0.175–3.76	0.789

*OR* odds ratio, *CI* confidence interval, *POAG* primary open angle glaucoma, *EXG* exfoliation glaucoma, *IOP* intraocular pressure, *KDB* Kahook Dual Blade, *phaco* phacoemulsification

High IOP on day 1 (OR = 1.1, 95% CI = 1.03–1.17) was also a risk factor for transient IOP elevation. Older patients (OR = 0.948, CI = 0.901–0.997) showed a lower risk of IOP spike occurrence.

## Discussion

In this study, we retrospectively followed the effects of KDB trabeculotomy in patients with POAG and EXG for six months, including the differences between KDB trabeculotomy alone and in combination with cataract surgery. In all four groups (EXG-alone, EXG-combined, POAG-alone, and POAG-combined), the IOP was decreased, the number of medications used was reduced, and postoperative IOP variability was greater with a standalone procedure than with a combined procedure.The risk of high IOP after surgery was higher in patients with a high IOP on postoperative day 1 and in patients who took more glaucoma medications before surgery; we also found a lower risk in older patients and in pseudophakic eyes.

Trabeculotomy ab interno is considered to have fewer complications than filtration surgery [4]. The risk of hypotony after trabeculotomy ab interno is very rare unless a cyclodialysis cleft occurs [11]. On the other hand, IOP spikes and increases have been reported to occur in 7.9–19.0% of patients undergoing trabeculotomy ab interno using a KDB [6, 7, 9, 12, 13]. In patients with a high IOP that persists after trabeculotomy ab interno, visual field defects may progress, or filtration surgery may be needed. Therefore, this study highlights the factors associated with IOP elevation and spikes after trabeculotomy ab interno using a KDB.

A procedure with concurrent phaco may facilitate the IOP-lowering effect of phaco alone [14], and the effect on IOP reduction is greater in eyes with pseudoexfoliation materials than in POAG eyes [15]. Sieck et al. reported that KDB trabeculotomy combined with cataract surgery decreases IOP more than KDB trabeculotomy alone [6]. Our results also showed that EXG-combined group achieved the lowest IOP among the EXG and POAG groups, and KDB-phaco the decreased a risk of postoperative IOP≥20 mmHg (Tables 4, 5 and 6) which is consistent with these reports. However, Kaplan–Meier analysis of the onset of IOP≥20 mmHg with or without previous phaco showed that eyes with previous phaco significantly failed to achieve rapid postoperative IOP reduction (supplementary Fig. 1, *p* = 0.0427, log-rank test). In addition, the groups demonstrated an age bias; the KDB-alone group (*n* = 63, mean age = 75.98) included 33 pseudophakic eyes from patients with a higher mean age (80.99) than the KDB-combined group (mean ages = 77.32). Therefore, further prospective studies with more cases and less group biases are needed.

Blood reflux is an important sign of correct incision location, but IOP occasionally increases due to the associated hyphema. Our logistic analysis showed that hyphema was not correlated with IOP elevation (Table 6).

Although the use of steroids sometimes elevates IOP, steroids are essential for supressing the formation of PAS that causes occlusion of the TM pathway after surgery. Chen et al. reported that topical corticosteroids increased the incidence of IOP spikes (defined as higher IOP levels than the preoperative IOP levels) within 3 months after gonioscopy-assisted transluminal trabeculotomy [16]. With regard to our method of steroid administration, whether shortening the duration of steroid drop treatment after surgery or changing to a weaker titre to reduce the risk of postoperative IOP elevation warrants further examination.

We stopped all hypotension drugs immediately after surgery to determine the effects of the surgery. Therefore, the greater the number of glaucoma medications that were used at baseline, the greater the impact of discontinuation, which may be the reason the number of preoperative medications was a risk factor for postoperative high IOP (Table 6). The time of resumption was mostly in the early postoperative phase (Tables 4 and 5), which may be associated with less variability in IOP levels at 3 and 6 months (Fig. 1). In addition, postoperative glaucoma eye drops were restarted based on a comprehensive assessment of the degree of visual field impairment and IOP level; however, there were no criteria for selecting the type of medication in this study. Future studies are needed to determine the appropriate timing for restarting medication and identifying the most effective drugs for controlling postoperative IOP.

Notably, we found that IOP on day 1 after surgery was a risk factor for postoperative IOP≥20 mmHg and transient IOP elevation≥20 mmHg (Table 6). The mechanism of IOP reduction by KDB trabeculotomy involves removal of the TM, which allows the aqueous humour to flow directly into Schlemm’s canal. Battista et al. reported that herniation of the TM into collector channels is associated with high IOP levels [17]. Thus, eyes in which IOP did not decrease immediately after surgery may exhibit anatomical abnormalities in the AH outflow pathway posterior to the TM.

In this study, in addition to the limitation of being a retrospective observation study, there were the following biases among the four groups (Table 2). 1) The mean age of patients with EXG was older than that of patients with POAG. 2) The preoperative IOP level was higher in EXG patients than in POAG patients. 3) The number of pseudophakic eyes was greater among EXG patients than among POAG patients. These biases are factors associated with postoperative IOP elevation (Table 6) and may have influenced the differences in KDB trabeculotomy efficacy in patients with EXG and POAG.

## Conclusions

KDB trabeculotomy is effective for patients with EXG and POAG, but postoperative IOP elevations must be considered, especially in patients with high IOP levels on day 1 after surgery and in patients who receive more glaucoma medications before surgery. A long-term prospective analysis is needed to identify additional factors associated with surgical outcomes in the future.

## Supplementary Information

**Additional file 1: Supplementary Figure S1.** Kaplan–Meier curve with or without a history of cataract surgery for the onset of IOP≥ 20 mmHg after surgery. Black line indicates eyes that did not have a history of cataract surgery, and red line indicates eyes with previous cataract surgery. Dotted lines indicate the 95% confidence intervals.

## Data Availability

The raw data is provided by the corresponding author upon reasonable request.

## Abbreviations

KDB: Kahook dual blade
KDB trabeculotomy: Trabeculotomy ab interno using a Kahook dual blade
EXG: Exfoliation Glaucoma
POAG: Primary Open Angle Glaucoma
IOP: Intraocular Pressure
TM: Trabecular Meshwork
ORs: Odds Ratio
phaco: Phacoemulsification
