# Supplementary figures and images for: Risk factors for intraocular pressure elevation in a six-month period after ab interno trabeculotomy using a Kahook Dual Blade
Source: BMC Ophthalmol. 2022 Jul 30;22:327. doi: 10.1186/s12886-022-02545-1 (PMC9338461; doi:10.1186/s12886-022-02545-1)

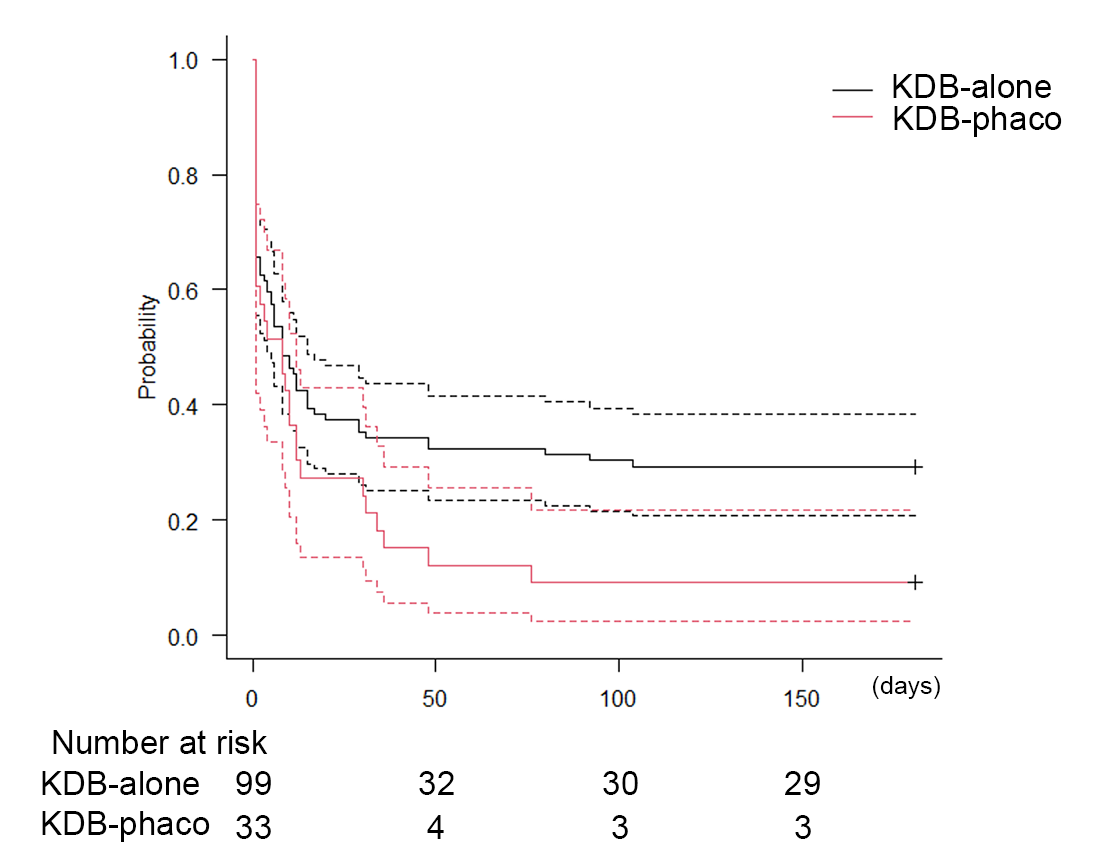

Supplement: Supplementary file 1 — Additional file 1: Supplementary Figure S1. Kaplan–Meier curve with or without a history of cataract surgery for the onset of IOP≥ 20 mmHg after surgery. Black line indicates eyes that did not have a history of cataract surgery, and red line indicates eyes with previous cataract surgery. Dotted lines indicate the 95% confidence intervals. [file 12886_2022_2545_MOESM1_ESM.tif]
